# Diagnostic accuracy of tests for type 2 diabetes and prediabetes: A systematic review and meta-analysis

**DOI:** 10.1371/journal.pone.0242415

**Published:** 2020-11-20

**Authors:** Gunjeet Kaur, P. V. M. Lakshmi, Ashu Rastogi, Anil Bhansali, Sanjay Jain, Yot Teerawattananon, Henna Bano, Shankar Prinja

**Affiliations:** 1 Department of Community Medicine and School of Public Health, Post Graduate Institute of Medical Education and Research, Chandigarh, India; 2 Department of Endocrinology, Post Graduate Institute of Medical Education and Research, Chandigarh, India; 3 Department of Internal Medicine, Post Graduate Institute of Medical Education and Research, Chandigarh, India; 4 Saw Swee Hock School of Public Health, National University of Singapore, Singapore, Singapore; 5 Health Intervention Technology Assessment Program, Nonthaburi, Thailand; Weill Cornell Medical College Qatar, QATAR

## Abstract

**Aim:**

This systematic review aimed to ascertain the diagnostic accuracy (sensitivity and specificity) of screening tests for early detection of type 2 diabetes and prediabetes in previously undiagnosed adults.

**Methods:**

This systematic review included published studies that included one or more index tests (random and fasting tests, HbA1c) for glucose detection, with 75-gram Oral Glucose Tolerance Test (or 2-hour post load glucose) as a reference standard (PROSPERO ID CRD42018102477). Seven databases were searched electronically (from their inception up to March 9, 2020) accompanied with bibliographic and website searches. Records were manually screened and full text were selected based on inclusion and exclusion criteria. Subsequently, data extraction was done using standardized form and quality assessment of studies using QUADAS-2 tool. Meta-analysis was done using bivariate model using Stata 14.0. Optimal cut offs in terms of sensitivity and specificity for the tests were analysed using R software.

**Results:**

Of 7,151 records assessed by title and abstract, a total of 37 peer reviewed articles were included in this systematic review. The pooled sensitivity, specificity, positive (LR+) and negative likelihood ratio (LR-) for diagnosing diabetes with HbA1c (6.5%; venous sample; n = 17 studies) were 50% (95% CI: 42–59%), 97.3% (95% CI: 95.3–98.4), 18.32 (95% CI: 11.06–30.53) and 0.51 (95% CI: 0.43–0.60), respectively. However, the optimal cut-off for diagnosing diabetes in previously undiagnosed adults with HbA1c was estimated as 6.03% with pooled sensitivity of 73.9% (95% CI: 68–79.1%) and specificity of 87.2% (95% CI: 82–91%). The optimal cut-off for Fasting Plasma Glucose (FPG) was estimated as 104 milligram/dL (mg/dL) with a sensitivity of 82.3% (95% CI: 74.6–88.1%) and specificity of 89.4% (95% CI: 85.2–92.5%).

**Conclusion:**

Our findings suggest that at present recommended threshold of 6.5%, HbA1c is more specific and less sensitive in diagnosing the newly detected diabetes in undiagnosed population from community settings. Lowering of thresholds for HbA1c and FPG to 6.03% and 104 mg/dL for early detection in previously undiagnosed persons for screening purposes may be considered.

## Introduction

In 2012, United Nation’s resolution titled “Future We Want” recognized diabetes as a priority disease under non-communicable diseases (NCDs) and a global challenge to sustainable development [1]. Owing to its growing burden across the globe, diabetes is also part of World Health Organization Global Action Plan for NCDs [2]. To this end, the Sustainable Development Goal 3.4 target envisions to achieve one-third reduction in premature mortality from the major NCDs including diabetes by year 2030 [3]. With the rising trajectory of diabetes worldwide, the International Diabetes Federation estimated that there would be 642 million people with diabetes by 2040 [4].

The cardinal characteristic of type 2 diabetes is chronic hyperglycaemia subsequent from shortcomings in either secretion or action of insulin, or maybe both. Further, prediabetes characterized by impaired glucose tolerance (IGT) and/or impaired fasting glycaemia (IFG), is considered as a risk category that may progress to diabetes and cardiovascular disease (CVD) [5]. Diabetes may also lead to microvascular and macrovascular complications that can have effect on eyes, kidney, nerves, feet and heart. The main drivers of this rising type 2 diabetes are associated with rapid urbanization and inadequate or lack of physical activity due to transitions in lifestyles [4, 6]. Nevertheless, type 2 diabetes not only has an effect at individual level, but due to chronic nature of the condition has implications at health system and economic level as well [7].

Globally, cost of diabetes including its related complications was US$ 548 billion in 2013 [8]. Estimates indicated that a person with diabetes utilized twice as much resources than with non-diabetes and experienced higher catastrophic health spending 17.8% (people with diabetes) vs. 13.9% (people with no-diabetes); (95% C.I. 0.2–7.7; p = 0.05) [8]. Moreover, this increasing prevalence of diabetes with associated complications may contribute to increase in healthcare costs [6]. Undeniably, the direct costs (including diabetes treatment and complications) and indirect costs arising from productivity losses are huge [9]. Approximately one-fifth of worldwide health spending in case of diabetes is being spent in the economies of low- and middle-income countries [10]. Majority of these health systems are oriented towards provision of acute care and thus insufficiently organized for providing for long term conditions of chronic care of non-communicable disease (NCD) [7].

Thus rising burden of type 2 diabetes, its long asymptomatic period, long term and short-term complications of the disease are adding on to increasing resource strain on health systems [7, 11]. In such an instance, promoting health interventions such as lifestyle modifications are few of the many criteria that appropriate for public policy support for screening of diabetes including pre-diabetes [12]. Moreover, diabetes fulfils the seven screening criteria under the widely used Wilson-Jugner criteria 1968 [13] for suitability to be part of screening programs. The benefits of screening for diabetes on mortality are not directly proven [14]. However, indirect benefits of screening may involve early detection of condition in apparently well individuals. This early detection of the condition may lead to lesser or delayed incidence of complications than those who were routinely diagnosed [15].

Across the globe, most of the screening programs for diabetes and prediabetes employed questionnaires/risk scoring tools and tests namely fasting blood/plasma glucose (FBG/FPG), HbA1c and random blood glucose (RBG) [5]. However, a systematic review by Engelgau summarized that risk scores do not perform well as stand-alone tests in screening programs and use of biochemical tests was encouraged [11]. The present guidelines adopted the cut off of HbA1c as 6.5% based on the findings of DETECT-2 study [16]. Further the International Expert Committee report also concluded that for identifying people at risk of developing complication like retinopathy, HbA1c 6.5% level provided sufficiently sensitive and specific evidence to capture the same [17]. There have been previous attempts to report on diagnostic accuracy of these blood tests [18, 19]. A systematic review by Bennet in 2007 narratively presented the findings for HbA1c for diabetes and did not undertake meta-analysis [18]. A meta-analysis by Kodama in 2013 included studies using abnormal A1c and FPG values for diagnosing and predicting diabetes [20]. Using data from previous two systematic reviews [18, 20], a meta-analytical comparison of HbA1c and FPG was done by Hoyer in 2018 [21]. Another published meta-analysis reported on the summary estimates for diagnostic accuracy for HbA1c for prediabetes [19]. However, little information is available about diagnostic accuracy of these most commonly used tests compared with a common reference standard for detection of type 2 diabetes and pre-diabetes in previously undiagnosed population. We aimed to bridge this gap in evidence by undertaking this systematic review. The main objective of this review was to assess the diagnostic accuracy (sensitivity and specificity) of screening tests for early detection of type 2 diabetes and prediabetes in individuals not previously diagnosed with diabetes. Our specific objectives focussed on summarising the evidence for various types of screening tests used to detect blood glucose levels; and determining the optimal cut-offs in terms of sensitivity and specificity for these tests from the evidence collated. Our findings may be useful to clinicians, health care managers and policy-makers involved in provision of health care for diabetes and prediabetes worldwide.

## Methods

The present systematic review is reported based on PRISMA-DTA checklist [22] and Meta-analysis and guided by “Cochrane Handbook for Systematic Reviews of Diagnostic Test Accuracy Reviews [23]. It was registered on the International prospective register of systematic reviews PROSPERO with CRD ID CRD42018102477.

### Eligible studies

We sought studies that reported the diagnostic accuracy of blood glucose tests for detecting type 2 diabetes (T2DM) and/or prediabetes in adults aged 18 years or more, recruited from community settings and without any previous history of type 2 diabetes. A study was considered also eligible if the study population below 18 years was ten per cent or less of that study population. Based on previous knowledge through a review of literature [5], the tests (venous or capillary sample) considered for screening for type 2 diabetes were random blood/plasma glucose, fasting blood/plasma glucose, HbA1c and post prandial glucose. 75-gram Oral Glucose Tolerance Test (or 2-hr post load glucose through venous route) was taken as the reference standard [24]. Studies where reference standard sample was taken through capillary route were not included. No restrictions on study design, time period or language were considered while carrying out the searches. Studies with index test and reference standards performed on all participants were considered. The studies using World Health Organization (WHO) or American Diabetes Association (ADA) or both criteria for diagnosis of diabetes & prediabetes were considered. Any opinion-piece, editorial, studies conducted in children, adolescents or pregnant women with type 2 diabetes, type 1 diabetes or in animals were excluded. Any study in non-English language was only excluded at time of analysis if English translation from either author of included studies or web/internet sources was unavailable. Case control studies were excluded as these studies are prone to bias [25].

### Data sources and searches

Search strategies were developed (S1 Appendix in S1 File) and modified accordingly to examine electronic databases from their inception to July 7, 2018. We updated the searches till 9^th^ March 2020. These databases were MEDLINE (OVID), Pubmed, EMBASE, Web of Science Core Collection (1952 till March 2020), CINAHL, Scopus and Cochrane (Cochrane Database of Systematic Reviews and Cochrane Central Register of Controlled Trials). The other sources like bibliographic searches of the relevant reviews identified during the screening and websites of World Health Organization and International Diabetes Federation were also searched for relevant records. Combinations of Medical Subject Heading terms (where applicable) and text words were employed to make search algorithm that was combined using Boolean operators. Specifically, terms (and their synonyms) to identify adults, index tests (Glycated Hemoglobin/HbA1c, fasting glucose, random glucose), reference standard (Oral Glucose Tolerance test), diabetes, prediabetes and outcomes like sensitivity and specificity were included in the search strategy (S1 Box in S1 File). The duplicates were removed automatically using Endnote Version X8 and manually during the screening.

### Study selection

Two reviewers (GK and HB) independently carried out the searches, manually screened and selected the records based on pre-decided inclusion and exclusion criteria. Further, the data was extracted using a standardized form. Further, disagreements at any stage of this systematic review were resolved by discussion with third reviewer (PVML) as arbitrator.

### Data extraction and quality assessment

Two reviewers independently extracted information using a data extraction form and further did quality assessment of included studies. Information on study setting, year of publication, sample size, prevalence of the target condition, methods of testing used, route of sample, reference standard were sought. Further, the data on diagnostic accuracy (sensitivity and specificity) were extracted by comparing the index tests against the reference standard for all the cut offs reported in the included studies. We included the information that was either provided in the study or we derived the number of true positives, false positives, false negatives and true negatives to generate two by two tables for respective cut-offs.

For the quality assessment, each included study was assessed using the Quality Assessment of Diagnostic Accuracy Studies (QUADAS-2) tool [26]. This tool has four domains comprising of patient selection, index test, reference standard and flow and timing under risk of bias assessment. The concerns regarding the applicability are ascertained for three domains of patient selection, index test and reference standard. The signaling questions to a domain were modified based on the review question and inclusion criteria. We did not consider the signaling question related to case control design being avoided in patient selection and whether all patients received reference standard in flow and timing domain. This was done in accordance with exclusion criteria decided. In order to rate quality (low, unclear, high) to a particular domain, we referred to the guiding points reported elsewhere [27]. If a study scored unclear or high for one or more signaling questions in a domain, then the domain was scored unclear/high risk of bias.

### Data synthesis and analysis

We undertook descriptive analysis to report on the number of studies by methods, year and country of publication, condition being diagnosed, and guidelines used for diagnosis of diabetes/prediabetes. Moreover, the included studies were tabulated by the index tests and reference standards. We undertook quantitative synthesis for the included studies that used the same index test with similar route of sample collection. We then pooled results based on a single data point from each study, and also with regard to the most commonly reported threshold as per the WHO and ADA guidelines for diabetes/prediabetes. We used a bivariate model to pool our data [23]. We used metandi command in STATA (version 14, STATACORP) to undertake meta-analysis; where a minimum of four or more studies was available for that particular test with same cut-off. We obtained summary estimates of sensitivity, specificity, positive and negative likelihood ratios (LR+ and LR-), with 95% confidence interval (CI). In order to calculate the optimal thresholds for the index test/s, we employed the novel approach and R code given by Steinhauser 2016 [28] for a continuous bio-marker that used 2×2 tables from multiple thresholds per study included in the meta-analysis. This was done using R software (package diagmeta) [29]. Further, the GRADEPro tool [30] was used for assessing the certainty of evidence collated for reporting on the optimal thresholds for the index test at the outcome level [31]. The prevalence or the pre-test probability was calculated for each included study in the meta-analysis and the median prevalence estimate with interquartile range was used in the GRADEPro tool [32]. Assessment of four domains in the GRADE program was done based on the available guidance documents [32–34] and the explanation was provided in the footnotes (S4 Table in S1 File). A “high”, “moderate”, “low” or “very low” level for certainty of the evidence for the recommendation was decided as per the number of domains satisfied [35].

## Results

### Screening and selection of literature

**Fig 1:**
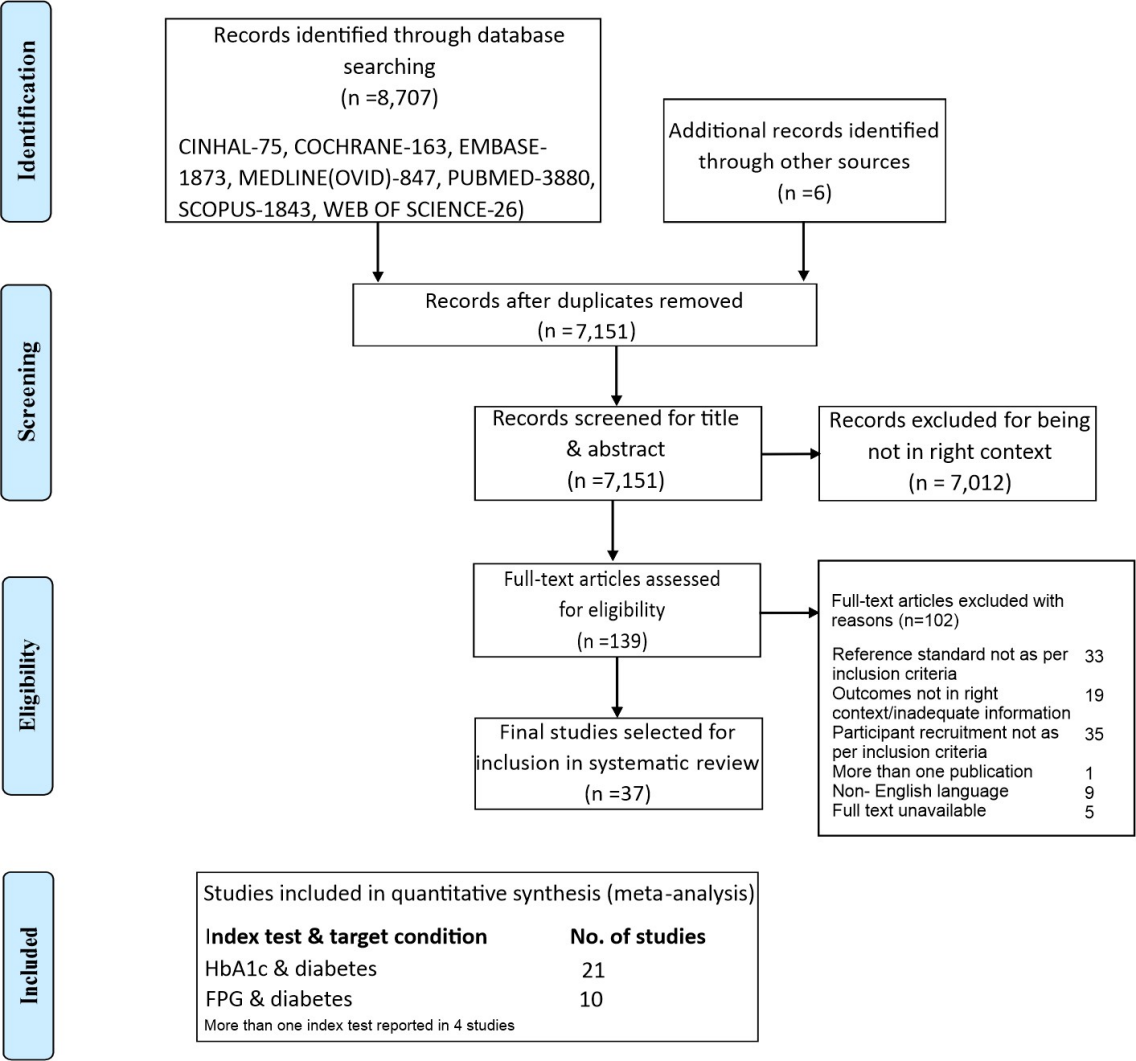
PRISMA flow diagram.

Fig 1 shows the detailed study selection process based on PRISMA-DTA reporting guidelines [36]. All the searches yielded a total of 8,713 records. After removal of duplicates (n = 1,562) and subsequent to title and abstract screening, thirty-seven studies were considered for the final selection. In case of insufficient information or non-English articles, the corresponding authors were contacted through electronic mail; however only studies with adequate information were included in the review. Of the 37 studies, 21 studies assessed only HbA1c test (12 for diabetes alone; 8 for diabetes and prediabetes; 1 for prediabetes alone); nine studies assessed FPG primarily for diabetes; four studies assessed both HbA1c and FPG (3 for diabetes alone; 1 for diabetes and prediabetes), two studies assessed fasting capillary glucose and one study assessed random capillary blood glucose.

### Characteristics of the included studies

A total of 1,07,534 participants (n = 25 studies) for diabetes; 39,846 for both diabetes and prediabetes (n = 11 studies); and 667 for prediabetes alone (n = 1 study) were included in this systematic review. Most studies were conducted in China (30%), USA (11%) followed by South Africa (8%) (S2 Box in S1 File).

For diagnosing diabetes/prediabetes, 44% (n = 16) studies used WHO guidelines, 42% (n = 15) used ADA guidelines; and 14% (n = 5) used both. The key characteristics of the included studies can be seen in Table 1.

**Table 1:** Key characteristics of included studies.

Author	Country of study	Year of publication	Target Condition	Sample analysed (N)	Diagnosis Criteria used	Blood glucose Test	Prevalence* (%) of diabetes with reference standard	No. (n) of diabetes diagnosed with reference standard	Prevalence of prediabetes based on reference standard	No. of prediabetes based on reference standard
Little [51]	USA	1988	Diabetes	381	WHO	HbA1c	34	112	-	-
Husseini [52]	Norway	2000	Diabetes	445	WHO	FCBG	2.7	12	-	-
Rodriguez-Moran [53]	Mexico	2001	Diabetes	712	ADA	FPG	9.12	65	-	-
Daniel [54]	Australia	2002	Diabetes	3249	ADA & WHO	FPG	11.6	377	-	-
Mannucci [45]	Italy	2003	Diabetes	1215	WHO	FPG		80	-	-
Nakagami [55]	Multi-country	2002	Diabetes	17512	-	FPG	6	1051	-	-
Al Lawati [56]	Oman	2007	Diabetes	4917	ADA & WHO	FPG	9.9	489	-	-
Somnnavar [57]	India	2009	Diabetes	1333	WHO & ADA	RCBG	13.9	185	-	-
Zhou [40]	China	2010	Diabetes & prediabetes	903	WHO	HbA1c	11.1	100	22.4	202
Araneta [58]	Japan	2010	Diabetes	933	ADA	HbA1c	15.5	145	-	-
Kramer [59]	Brazil	2010	Diabetes	2107	ADA	HbA1c		198	-	-
Mohan V [22]	India	2010	Diabetes	2188	WHO	HbA1c	10.1	220	-	-
van’t Riet [60]	Netherlands	2010	Diabetes	2753	WHO	HbA1c	4	107	-	-
Choi [61]	Korea	2011	Diabetes	9375	ADA	HbA1c	6.8	635	-	-
Bhowmik [41]	Bangladesh	2013	Diabetes & prediabetes	2293	WHO	HbA1c	7.9	181	8.6	197
Zhao [62]	China	2013	Diabetes & prediabetes	993	WHO	FCG	5.7	57	14.6	145
Wu [63]	China	2013	Diabetes & prediabetes	3354	WHO	HbA1c	21.26	725	40.16	1347
Ma Hui [64]	China	2013	Diabetes & prediabetes	1973	WHO	HbA1c	13.7	271	24	474
Huang [43]	China	2013	Diabetes	6540	ADA	HbA1c	6.04	422	-	-
Vlaar [65]	Netherlands	2013	Diabetes & prediabetes	944	ADA	HbA1c	3.7	35	20.2	191
Liang [66]	China	2014	Diabetes & prediabetes	8239	WHO	HbA1c	10.7	880	19	1565
Huang [67]	USA	2015	Diabetes	5782	ADA	FPG	-	231	-	-
Aekaplakorn [68]	Thailand	2015	Diabetes & prediabetes	6884	ADA	FPG	-	759	-	
Zemlin [69]	South Africa	2015	Prediabetes	667	ADA	HbA1c	-	-	27.7	185
Bao [70]	China	2015	Diabetes & prediabetes	7464	WHO & ADA	FPG	-	282	9	-
Incani [71]	Italy	2015	Diabetes & prediabetes	462	ADA	HbA1c	11	51	65	300
Aviles Santa [72]	USA	2016	Diabetes	15507	ADA	HbA1c	4.4	764	-	-
Hird [42]	South Africa	2016	Diabetes	1077	WHO	HbA1c	3.5	38	-	-
Karnchanasorn [73]	USA	2016	Diabetes	5764	ADA	HbA1c	6.8	392	-	-
Liu [74]	China	2016	Diabetes & prediabetes	7611	WHO	HbA1c	-	411	-	473
Zou [75]	China	2016	Diabetes	3050	WHO	HbA1c	10.2	311	-	-
Herath [76]	Sri Lanka	2017	Diabetes	254	ADA & WHO	HbA1c	16.1	41	-	-
Wu [77]	China	2017	Diabetes	4325	WHO	HbA1c	13.8	-	-	
Zhou [78]	China	2018	Diabetes & prediabetes	7909	WHO	HbA1c	8.79	695	19.1	1514
Lim [79]	Singapore	2018	Diabetes	3540	ADA	HbA1c & FPG	-	332	-	-
Prakashchandra [80]	South Africa	2018	Diabetes	1378	ADA	HbA1c & FPG	-	154	-	-
Katulanda [81]	Sri Lanka	2019	Diabetes	4014	ADA	FPG	4.7	191	-	-

* Prevalence or number of participants with diabetes based on OGTT/2hrPG values.

### Quality assessment

Of the total twenty-three studies that employed HbA1c by blood sample/venous route, fourteen studies scored unclear risk of bias in the section on patient selection. Inadequate information on sampling methods (consecutive/random) employed was the prime cause (S2 and S3 Figs in S1 File). Further, one study scored unclear risk in same index test. Studies that mentioned description of diagnostic criteria to diagnose diabetes/prediabetes or on methods of sample collection for index test/reference standard were given low risk of bias. In addition, four studies were assigned unclear risk in flow and timing because inadequate information or longer duration between index and reference test could have introduced bias. One out of two studies using HbA1c by capillary route scored unclear risk of bias in patient selection, index test and reference standard (S3 and S4 Figs in S1 File). For studies that assessed FPG test (n = 13), six studies were assigned unclear risk in patient selection, index test and reference standard domains. Those studies (n = 2) where test accuracy results were not reported separately by 2-hrPG OGTT were given as unclear risk; this was due to uncertainty in interpretation of results of FPG without knowledge of result of OGTT as FPG is also part of the latter (S5 and S6 Figs in S1 File). For applicability concerns, most of the studies for all tests were treated as low concern.

### Pooled diagnostic accuracy of blood glucose tests (meta-analysis)

**Fig 2:**
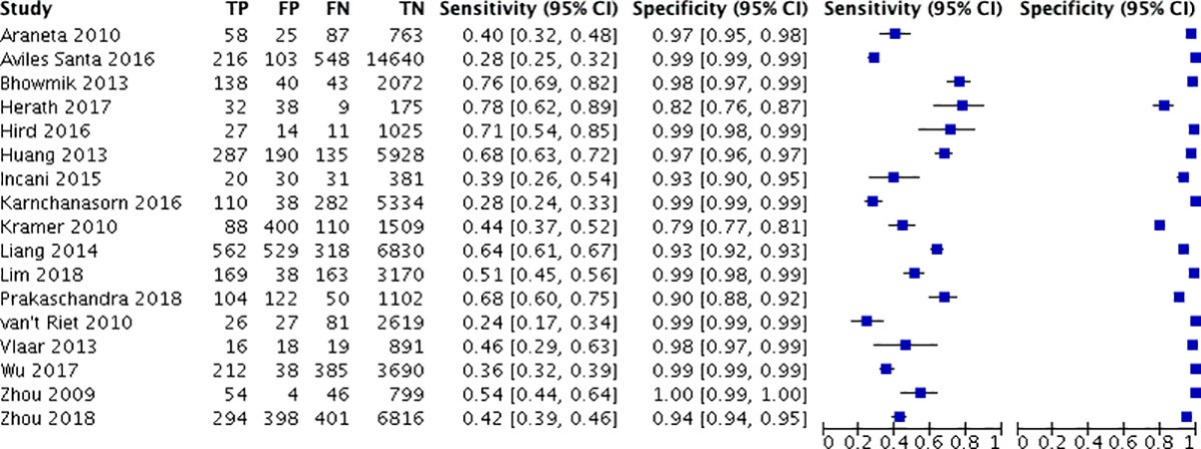
Forest plot of HbA1c 6.5% for detecting diabetes.

**Fig 3:**
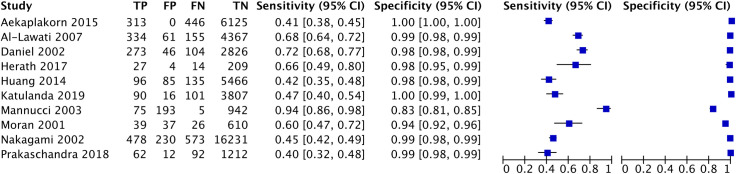
Forest plot of FPG 126 mg/dL for detecting diabetes.

**Fig 4:**
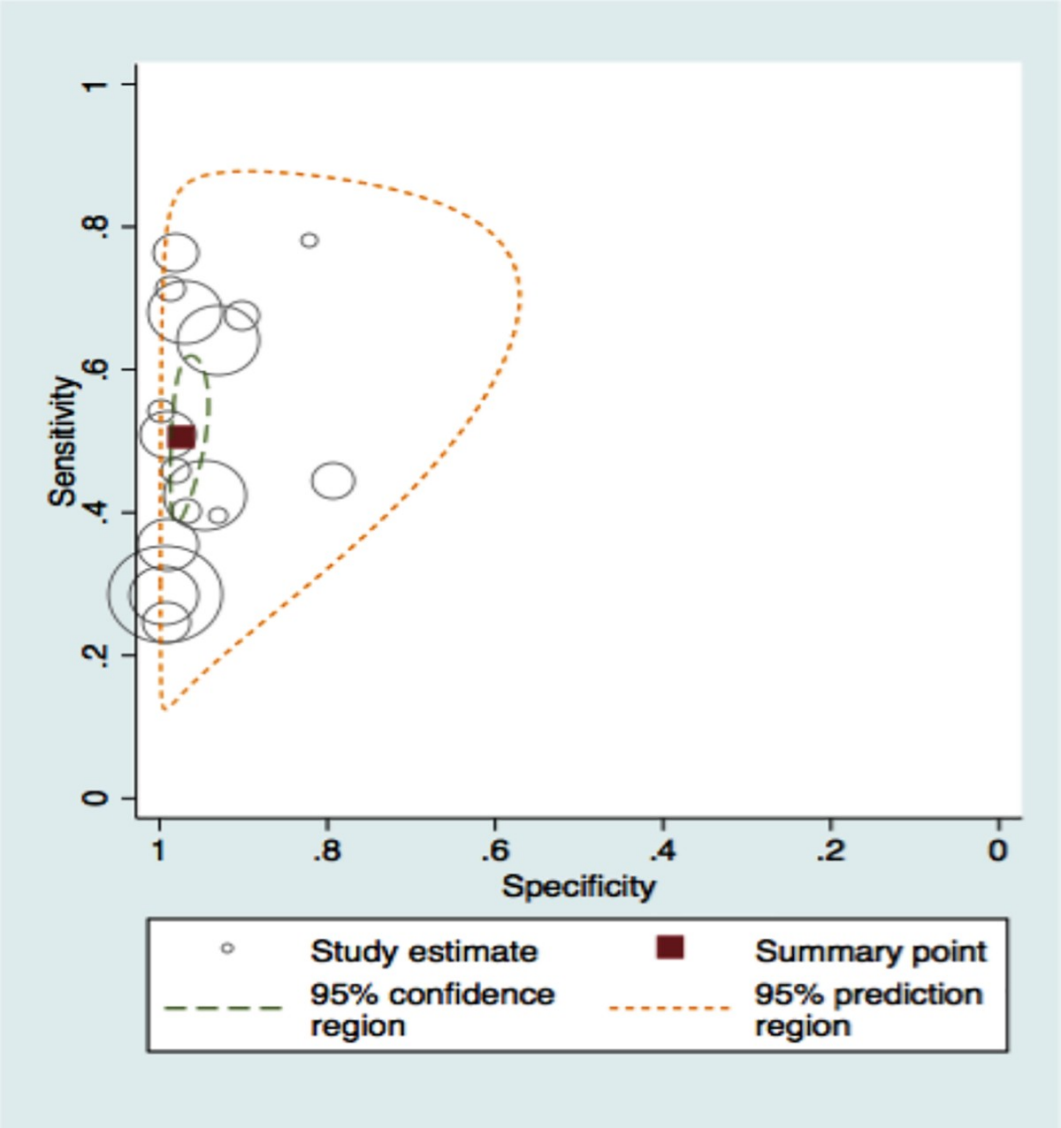
Summary receiver operating characteristic plot of HbA1c (6.5%) for detecting diabetes.

**Fig 5:**
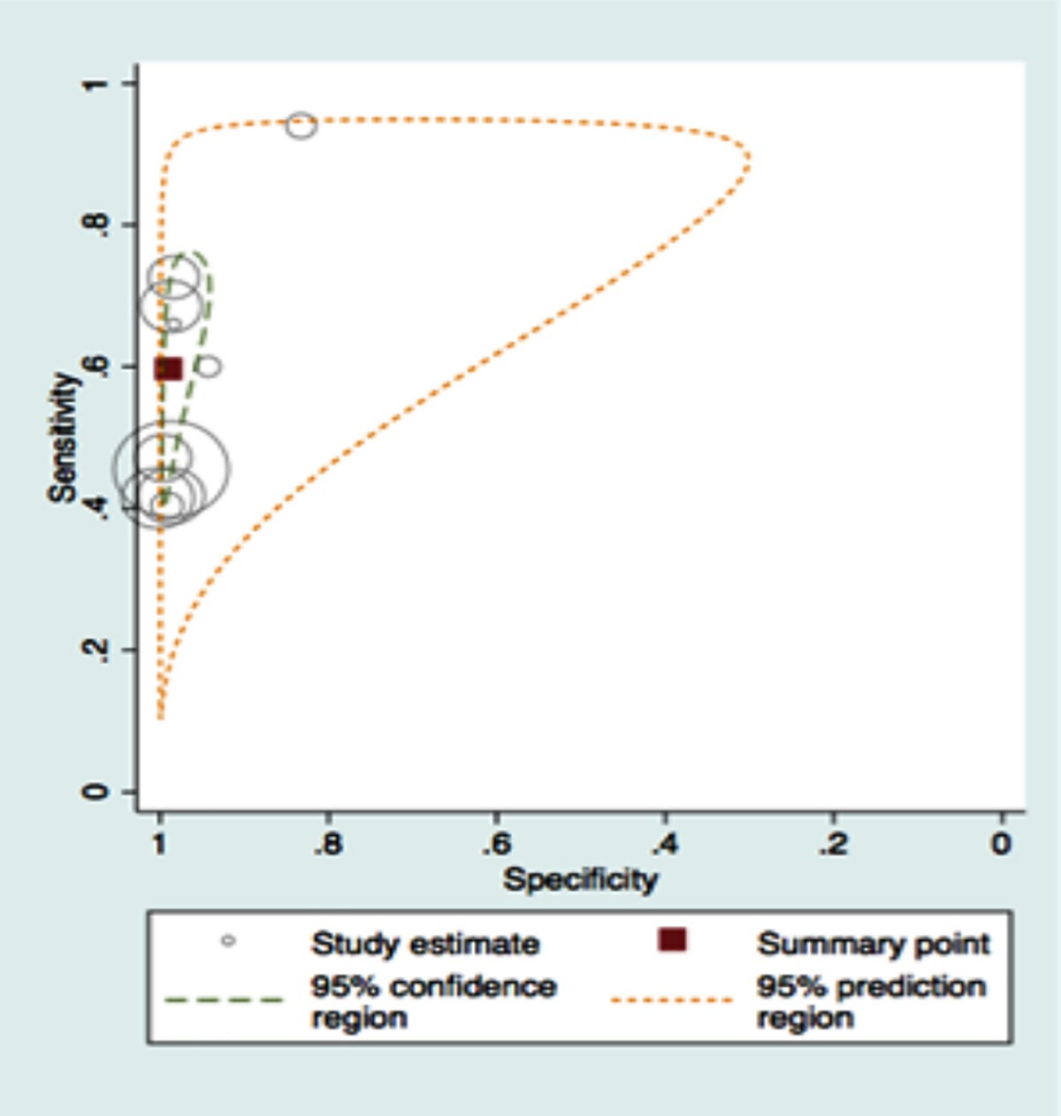
Summary receiver operating characteristic plot of FPG (126 mg/dL) for detecting diabetes.

A total of twenty-one studies were included in meta-analysis for HbA1c and ten studies for FPG for diabetes for various thresholds with the number of studies included and cases, their combined sensitivities and specificities shown in Table 2. The number of true positives and negatives and false positives and negatives at the recommended thresholds of HbA1c 6.5% and FPG 126mg/dl are depicted in forest plots in Figs 2 and 3 respectively. The summary sensitivity, specificity, LR+ and LR- for HbA1c at a common cut off of 6.5% (venous sample) for diagnosing diabetes were 50% (95% CI: 42–59%), 97% (95% CI: 95–98%), 18.3 (95% CI: 11–30) and 0.51 (95% CI: 0.432–0.605), respectively. Details of stratified analysis with studies using HbA1c against OGTT and with WHO criteria are provided in S2 Table in S1 File. Similarly, for the FPG test (cut off as 126 mg/dL) the corresponding values are 59.4% (95% CI: 46.6–71%), 98.8% (95% CI: 96.5–99.6%), 47.825 (95% CI: 19.10–119.73) and 0.411 (95% CI: 0.305–0.555). Figs 4 and 5 show the SROC plots for these two tests HbA1c (6.5%) and Fasting Plasma Glucose (126 mg/dL) for diabetes respectively.

**Table 2:** Pooled estimates (meta-analysis) at various cut-offs for diagnostic accuracy of HbA1c (%) and FPG (mg/dL) for diabetes.

	Threshold value used for diabetes	Number of studies	Number of cases (true positives & false negatives) & participants	Sensitivity (95% CI)	Specificity (95% CI)	Positive Likelihood ratio (95% CI)	Negative Likelihood ratio (95% CI)
**HbA1c (%)**	**5.7**	7	2506/29076	0.888 (0.830–0.927)	0.657 (0.531–0.765)	2.588 (1.878–3.566)	0.171 (0.119–0.246)
	**5.8**	8	3127/36863	0.818 (0.749–0.871)	0.781 (0.680–0.857)	3.738 (2.587–5.401)	0.233 (0.175–0.310)
	**5.9**	7	2958/34866	0.770 (0.6874–0.837)	0.834 (0.742–0.898)	4.644 (3.080–7.0022)	0.276 (0.209–0.363)
	**6.0**	10	3381/39115	0.757 (0.681–0.819)	0.893 (0.843–0.929)	7.084 (4.896–10.254-)	0.272 (0.208–0.356)
	**6.1**	7	2543/27679	0.726 (0.596–0.826)	0.932 (0.873–0.964)	10.605 (6.166–18.240)	0.294 (0.199–0.436)
	**6.2**	4	2118/23217	0.655 (0.538–0.7554)	0.935 (0.872–0.968)	10.042 (5.672–17.781)	0.370 (0.279–0.490)
	**6.3**	6	1710/17151	0.654 (0.574–0.727)	0.945 (0.902–0.970)	11.960 (6.940–20.610)	0.366 (0.297–0.450)
	**6.4**	5	2059/21670	0.624 (0.527–0.712)	0.950 (0.904–0.975)	12.589 (7.079–22.387)	0.396 (0.317–0.494)
	**6.5**	17	5132/64928	0.502 (0.417–0.588)	0.973 (0.953–0.984)	18.328 (11.067–30.353)	0.512(0.432–0.605)
**FPG (mg/dL)**	**126**	10	3438/45917	0.594 (0.466–0.710)	0.988 (0.965–0.996)	47.825 (19.104–119.729)	0.411 (0.305–0.555)

* Estimates are rounded off to nearest number or three decimal places.

**Fig 6:**
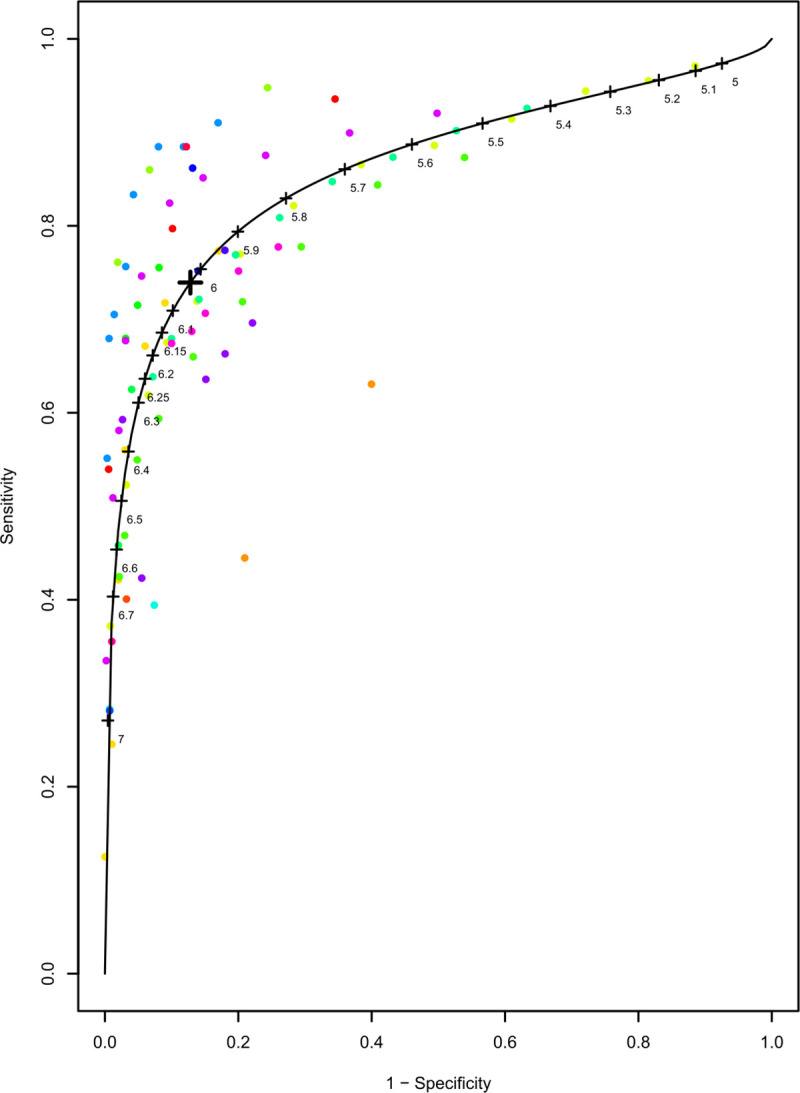
Summary receiver operating characteristic curve showing the optimal cut off of HbA1c 6.03% for detecting diabetes.

**Fig 7:**
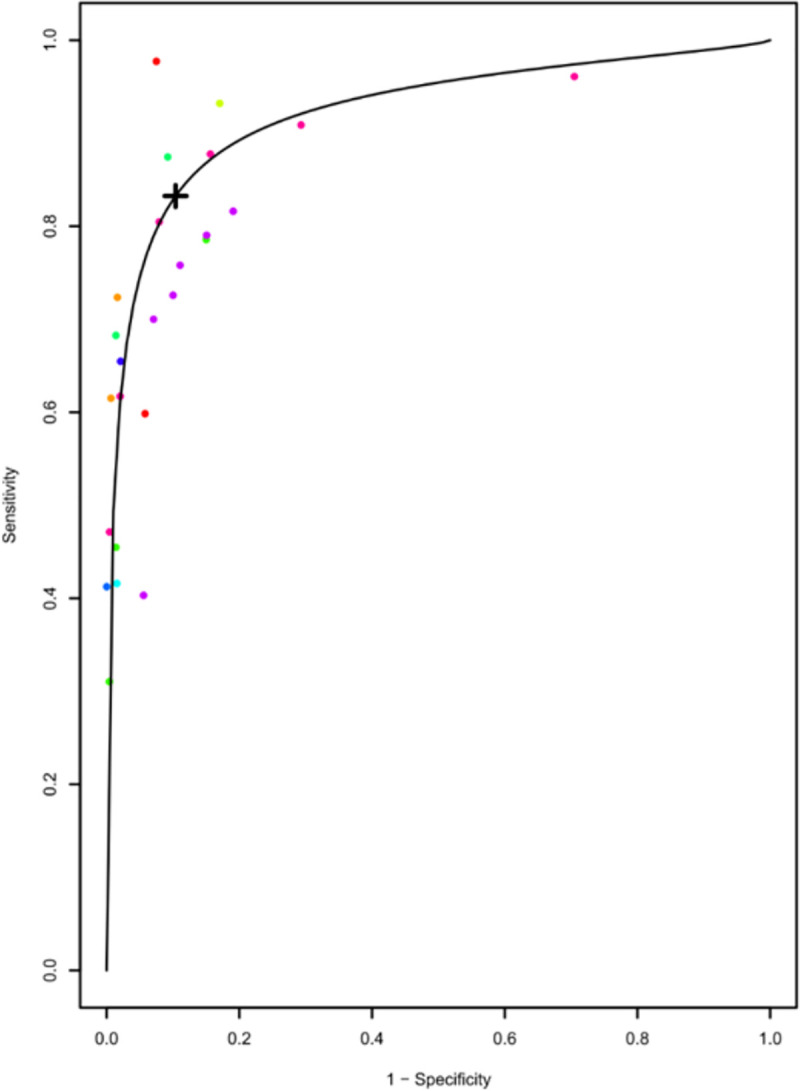
Summary receiver operating characteristic curve showing the optimal cut off of FPG 104 mg/dL for detecting diabetes.

The optimal cut off value for sensitivity and specificity for HbA1c for diagnosing diabetes in previously undiagnosed population was estimated as 6.03%. The pooled sensitivity and specificity at this optimal threshold for HbA1c 6.03% for diabetes were 74% (95% CI: 68–79%) and 87.2% (95% CI: 82–91%). Fig 6 shows this optimal cut-off for HbA1c on summary receiver operating characteristic curve; where each study is denoted by a coloured circle and numbers along the curve represent various thresholds for HbA1c. Estimated optimal cut-off for FPG for diagnosing diabetes was 104 mg/dL with pooled sensitivity of 82.3% (95% CI: 74.6–88.1%) and specificity of 89.4% (95% CI:85.2–92.5%) (Fig 7).

Using the GRADE approach, we found that the certainty of evidence collated at the outcome level (sensitivity and specificity) for optimal cut off of HbA1c 6.03% was of moderate quality (S5 Table in S1 File). The estimated median prevalence (with interquartile range) of diabetes from the included studies in the meta-analysis for HbA1c (n = 21) was 9.38% (IQR: 6.77–11.07).

## Discussion

This meta-analysis summarizes the evidence on paired outcomes (sensitivity and specificity) of diagnostic accuracy for the tests (HbA1c, FPG) used in the screening of diabetes and prediabetes in previously undiagnosed population. We found higher values of summary estimates specificity than sensitivity for both HbA1c and FPG at the common thresholds recommended by WHO and ADA guidelines for diagnosis of diabetes. The most relevant finding of our meta-analysis was determination of optimal thresholds of 6.03% for HbA1c and 104 mg/dL for FPG in previously undiagnosed population for detecting diabetes. However, there were insufficient number of studies that estimated diagnostic accuracy over the range of cut-offs to diagnose prediabetes as per present WHO/ADA guidelines (S6 Table in S1 File). So, we could not perform meta-analysis for the same.

This meta-analysis provides a comprehensive overview regarding diagnostic accuracy of these tests for an early diagnosis for diabetes in previously undiagnosed population. Based on the evidence collated from the test accuracy studies, the sensitivity and specificity ranged from 24% to 78% and 79% to 100% respectively for HbA1c (6.5%) for diagnosis of diabetes. Variation in sensitivity from 40% to 94% and specificity from 83% to 100% for FPG 126 mg/dl was noted. These are the two most frequently used blood glucose tests recommended for screening for type 2 diabetes across high income country settings [19].

Our findings in terms of estimates of pooled sensitivity for HbA1c 6.5% (pooled sensitivity-0.502) are slightly lower to those reported elsewhere in meta-analysis by Xu 2014 (pooled sensitivity—0.518) for Chinese adults [37]. However, our summary estimates of sensitivity are higher than those reported in another study (pooled sensitivity-0.371) that evaluated diagnostic test accuracy of HbA1c against 2hrOGTT[38]. On the contrary, our finding of pooled specificity for HbA1c 6.5% is higher than reported by Xu 2014 and lower than in [38]. Two other published systematic reviews did not undertake meta-analysis and narratively reported on diagnostic accuracy of HbA1c for diabetes screening [18, 39]. Moreover, the latter systematic review took into account both people with and without diabetes and reviewed performance of HbA1c for prediction of microvascular complications like retinopathy [39]. Our results found a lower sensitivity but slightly higher specificity for FPG (126mg/dl or 7mmol/l) detecting diabetes in undiagnosed persons than estimated by another meta-analysis [38]. Our finding of optimal cut-off of HbA1c as 6.03% for diagnosis of diagnosis in previously undiagnosed population lies within the range suggested by a previous work [18, 21]; and close to optimal cut-off (6.0%) estimated by a number of included cross-sectional studies [40–44]. We found the certainty of evidence for the optimal threshold for sensitivity and specificity for HbA1c (6.03%) as of moderate quality (S6 Table in S1 File). We downgraded by one level for risk of bias in patient selection. Methods of recruitment like through invitation or volunteering may lead to bias through self-referral unlike when random/consecutive sampling techniques are used. Similar observation has been reported previously [19, 45]. However, our finding of optimal threshold for FPG differs from that estimated by Hoyer 2018 [21].

Considering the rising prevalence of diabetes worldwide, our findings have important implications from both clinical and policy perspective. There is an ever-growing debate on the present cut-offs proposed for diagnosing diabetes and prediabetes [46]. HbA1c level values are indicator of long term glucose control and also provide a link to development of microvascular complications [46]. However, it is also true that the growing epidemic of diabetes warrants for tests with higher sensitivity for early identification of the disease. Thus, based on our review findings and previous work [21] lowering the thresholds for higher sensitivity for screening purpose may be considered. An early institution of preventive interventions for people at high risk and treatment control for newly diagnosed can help in reducing the incidence of complications in people with diabetes. It is noteworthy to mention here that the risk of complications like mortality risk from cardiovascular disease starts in the prediabetes stage even before clinical diabetes sets in and may also lead to significant morbidities as well [5, 47]. Similarly, people with diabetes are at about twice the risk of premature mortality than those without it [48]. Diabetes is also risk factor for other conditions like end-stage renal disease, retinopathy, peripheral vascular disease, cerebrovascular disease and other disabling conditions like depression. Development of complications magnify the cost of care for both the health provider and the individual.

There are several strengths of the present systematic review and meta-analysis. Firstly, a thorough search was done in all relevant electronic databases, irrespective of any filters based on time, design, country or language of records on diagnostic accuracy of the index tests specified. Secondly, the studies included are representative individuals (≥ 18 years) without any previously diagnosed diabetes, primarily recruited from community settings across the globe and of mixed ethnicities. Thirdly, only those studies were chosen wherein the index and reference standards were done on all the sampled population. Fourthly, we analysed and demonstrated the pooled estimates of diagnostic accuracy of the index tests with the use of bivariate random effects model, addressing inherent heterogeneity in these diagnostic accuracy studies. These random effects models are the most commonly recommended methods of synthesis for diagnostic accuracy meta-analysis [49]. These models have an advantage that, unlike previous methods, they account for both within-study and between-study variability [49]. Finally, our estimates of optimal cut-offs are based on a newer approach by Steinhauser 2016 reported elsewhere that makes use of all the available information reported on thresholds in case of continuous biomarkers and avoids any overestimation of results [28]. In general, while undertaking a meta-analysis for diagnostic accuracy, each study contributes only one pair of sensitivity and specificity. However, if studies present more than one threshold, as in our case, reducing the data and selecting a specific threshold per study to find out optimal cut-off may lead to inadequate use of information and thus introduce a bias. We incorporated all the information from the studies included in the meta-analysis to estimate optimal cut-off for the index tests.

Our present work had several limitations. Firstly, we ourselves did not undertake any further translations of the studies that were in non-English language. Secondly, no indirect comparisons between the different index tests to establish the best test for diagnosing diabetes and prediabetes were done. Thirdly, due to insufficient number of studies, the pooled estimates for prediabetes and other tests like random, fasting and HbA1c by capillary method could not be estimated in this review. Fourthly, we did not attempt to rate certainty of evidence for optimal cut-off FPG. This becomes challenging to implement and interpret especially when few studies report on multiple tests. Further guidance may be helpful to users on how to rate evidence when newer methods of pooling using multiple information are used. Lastly, we did not undertake sub-group analysis based on the ethnicity, classification of country region by income or methods. A systematic review [50] investigated the effect of ethnicity on HbA1c values in people without diabetes. However, exploring the role of ethnicity in estimation of optimal thresholds for these index tests and which is the best test to diagnose can be considered as future area of research. Further, the optimal cut-offs estimated for HbA1c and FPG are chiefly from statistical perspective. Role of clinical parameters and economic decision modelling for various screening strategies with these tests can be another future area of research.

In summary, our findings on the pooled estimates of diagnostic accuracy like sensitivity and specificity can be useful to researchers and policy makers for undertaking health technology assessments (HTA) for various screening strategies for diabetes. Lowering of thresholds of HbA1c to 6.03% or FPG to 104 mg/dl may be considered for screening for diabetes in previously undiagnosed individuals.

## Supporting information

S1 Checklist PRISMA DTA checklist. (DOCX)

S1 File (DOCX)
