# Supplementary material for: Diagnostic accuracy of tests for type 2 diabetes and prediabetes: A systematic review and meta-analysis
Source: PLoS One. 2020 Nov 20;15(11):e0242415. doi: 10.1371/journal.pone.0242415 (PMC7678987; doi:10.1371/journal.pone.0242415)
Supplement: S1 Checklist — (DOCX) [file pone.0242415.s001.docx]

**PRISMA DTA CHECKLIST (1)**

| **Section and Topic** | **Item No.^a^** | **Description** | **Page No.** |
| --- | --- | --- | --- |
| Title^b^ | 1 | Identify the report as a systematic review (meta-analysis) of DTA studies. | 1 |
| Abstract^b^ | 2 | A checklist for abstracts appears in Table 3. | 2 |
| Introduction | | | |
| Rationale^c^ | 3 | Describe the rationale for the review in the context of what is already known | 5-6 |
| Clinical role  of index test^d^ | D1 | State the scientific and clinical background, including the intended use and clinical role of the index test, and if  applicable, the rationale for minimally acceptable test accuracy (or minimum difference in accuracy for  a comparative design). | 5 |
| Objectives^b^ | 4 | Provide an explicit statement of question being addressed in terms of participants, index test, and target conditions | 6 |
| Methods | | | |
| Protocol and  Registration^c^ | 5 | Indicate where the review protocol can be accessed (eg, web address) and provide trial registration number if available. | 7 |
| Eligibility criteria^b^ | 6 | Specify study characteristics (participants, setting, index test, reference standards, target conditions, and study design)  and report characteristics (eg, years considered, language, publication status) used as criteria for eligibility and  providing rationale | 7 |
| Information sources^c^ | 7 | Describe all information sources (eg, databases with dates of coverage, contact with study authors to identify additional  studies) in the search and the date last searched. | 7-8 |
| Search^b^ | 8 | Present full search strategies for all electronic databases and other sources searched, including any limits used so that they can be repeated | Appendix S1 |
| Study selection^c^ | 9 | State the process for selecting studies (ie, screening, eligibility, whether included in systematic review, and, if applicable, included in the meta-analysis). | 8 |
| Data collection  Process^c^ | 10 | Describe the methods of data extraction from reports (eg, piloted forms, independently, in duplicate) and any processes  for obtaining and confirming data from the investigators. | 7-8 |
| Definitions for data Extraction^b^ | 11 | Provide definitions used in data extraction and classifications of target conditions, index tests, reference standards,  and other characteristics (eg, study design, clinical setting). | 8 |
| Risk of bias  and applicability^b^ | 12 | Describe methods used for assessing risk of bias in individual studies and concerns regarding the applicability to the review question | 8-9 |
| Diagnostic accuracy Measures^b^ | 13 | State the principal diagnostic accuracy measures reported (eg, sensitivity, specificity) and state the unit of assessment  (eg, per patient vs per lesion). | 9-10 |
| Synthesis of results^b^ | 14 | Describe the methods of handling the data, combining the results of the studies and describing the variability between studies. This could include, but I s not limited to (1) handling of multiple definitions of the target condition, (2) handling of multiple thresholds of test positivity, (3) handling multiple index test readers, (4) handling of indeterminate test results, (5) grouping and comparing tests, and (6) handling of different reference standards. | 9 |
| Meta-analysis^d^ | D2 | Report the statistical methods used for meta-analyses if performed. | 9 |
| Additional analyses^c^ | 16 | Describe the methods of the additional analyses (eg, sensitivity or subgroup analyses, meta-regression) if done, indicating which were prespecified. | - |
| Results | | | |
| Study selection^c^ | 17 | Provide the numbers of studies screened, assessed for eligibility, included in the review, and included in the meta-analysis if applicable, with reasons for exclusions at each stage, ideally with a flow diagram | 10 & Figure 1 |
| Study characteristics^b^ | 18 | For each included study, provide citations and present key characteristics including (1) participant characteristics (presentation, prior testing), (2) clinical setting, (3) study design, (4) target condition definition, (5) index test, (6) reference standard, (7) sample size, and (8) funding sources. | 10 & Table1 |
| Risk of bias and applicability | 19 | Present evaluation of risk of bias and concerns regarding applicability for each study. | 11 |
| Results of individual Studies^b^ | 20 | For each analysis in each study (eg, unique combination of index test, reference standard, and positivity threshold), report 2 × 2 data (TP, FP, FN, TN) with estimates of diagnostic accuracy and confidence intervals, ideally with a forest  plot or a receiver operating characteristic plot. | 12 & Table S3-S4 |
| Synthesis of results^b^ | 21 | Describe test accuracy, including variability; if meta-analysis was done, include results and confidence intervals. | 11-13; Table 2 |
| Additional analyses^b^ | 23 | Give results of additional analyses if done (eg, sensitivity or subgroup analyses, meta-regression, analysis of index test, failure rates, proportion of inconclusive results, and adverse events). | - |
| Discussion | | | |
| Summary^b^ | 24 | Summarize the main findings including the strength of the evidence. | 14-16 |
| Limitations^b^ | 25 | Discuss limitations from included studies (eg, risk of bias and concerns regarding applicability) and from the review  process (eg, incomplete retrieval of identified research). | 16 |
| Conclusions^b^ | 26 | Provide a general interpretation of the results in the context of other evidence. Discuss implications for future research and clinical practice (eg, the intended use and clinical role of the index test). | 16-17 |
| Other |  |  |  |
| Funding^c^ | 27 | For the systematic review, describe the sources of funding and other support and the role of the funders. | 17 |
|  |  |  |  |

**Reference**

1. McInnes MDF, Moher D, Thombs BD, McGrath TA, Bossuyt PM, Clifford T, et al. Preferred Reporting Items for a Systematic Review and Meta-analysis of Diagnostic Test Accuracy Studies: The PRISMA-DTA Statement. Jama. 2018;319(4):388-96.
