# Supplementary material for: Diagnostic accuracy of tests for type 2 diabetes and prediabetes: A systematic review and meta-analysis
Source: PLoS One. 2020 Nov 20;15(11):e0242415. doi: 10.1371/journal.pone.0242415 (PMC7678987; doi:10.1371/journal.pone.0242415)
Supplement: S1 File — (DOCX) [file pone.0242415.s002.docx]

**Supplementary File**

**Box S1: Keywords for Electronic Searches**

| - Adults [MeSH] - Middle-aged adults - Young adults - elderly - Random Blood (plasma) Glucose - Random Blood Glucose - Random Capillary Blood Glucose - RBG - RCBG - Capillary Blood Glucose - CBG - Fasting Plasma Glucose - Fasting Blood Glucose - FBG - Glycated Hemoglobin [MeSH] - Hemoglobin A, Glycosylated - Fasting Plasma Glucose - Fasting Blood Glucose - FBG | - Glycated Hemoglobin [MeSH] - Hemoglobin A, Glycosylated - HbA1c - HbA1 - Glycosylated Hemoglobin A - Hemoglobin, Glycosylated A1 - Glycated Hemoglobin A/analysis [MeSH] - Glycated Hemoglobin A/ standards [MeSH] - Oral Glucose Tolerance Test - OGTT - Glucose Tolerance Test - Diabetes Mellitus, Type 2 [MeSH] - Oral Glucose Tolerance Test - OGTT - Glucose Tolerance Test - Diabetes Mellitus, Type 2 [MeSH] | - Hyperglycemia/diagnosis [MeSH] - Diabetes, Type 2/blood - Prediabetic state - Pre-diabetic state - Prediabetes - Pre-diabetes - Impaired Fasting Glucose - IGT - IFG - Impaired Glucose Tolerance - Glucose intolerance/blood [MeSH] - Glucose intolerance/diagnosis [MeSH] - Sensitivity - Specificity - Sensitivity and Specificity [MeSH] - Predictive value of tests - ROC curve - ROC analys* |
| --- | --- | --- |

| **Appendix S1: SEARCH STRATEGIES** | |
| --- | --- |
| **Databases *** | **Results** |
| **Pubmed**  (((((((((((((adult[MeSH Terms]) OR (adult[Title/Abstract])) OR (adults[MeSH Terms])) OR (adults[Title/Abstract])) OR (frail adults[MeSH Terms])) OR (frail adults[Title/Abstract])) OR (young adults[MeSH Terms])) OR (middle-aged adults[MeSH Terms])) OR (middle-aged adults[Title/Abstract])) AND ((((((((("Random Capillary Blood Glucose"[Title/Abstract]) OR ("Random Blood Glucose"[Title/Abstract])) OR ("Capillary Blood Glucose"[Title/Abstract])) OR ("Fasting Blood Glucose"[Title/Abstract])) OR ("Fasting Plasma Glucose"[Title/Abstract])) OR ("FPG"[Title/Abstract])) OR ("Glycated Hemoglobin A"[MeSH Terms])) OR ("Glycated Hemoglobin A"[Title/Abstract])) OR ("Post Prandial Glucose"[Title/Abstract]))) AND ((((((((("Diabetes mellitus"[MeSH Terms]) OR ("Diabetes mellitus"[Title/Abstract])) OR ("Diabetes Mellitus, Type 2"[Title/Abstract])) OR ("Prediabetic State"[Title/Abstract])) OR (Prediabet*[Title/Abstract])) OR ("Impaired Glucose Tolerance"[Title/Abstract])) OR ("IGT"[Title/Abstract])) OR ("Impaired Fasting Glucose"[Title/Abstract])) OR ("IFG"[Title/Abstract]))) AND ((((((Sensitivity[Title/Abstract]) OR (Specificity[Title/Abstract])) OR ("Sensitivity and Specificity"[MeSH Terms])) OR ("predictive value of tests"[MeSH Terms])) OR ("predictive value of tests"[Title/Abstract])) OR ("ROC Curve*"[Title/Abstract]))) | 3,880 |
| **EMBASE**  Inception till present  ('adult'/exp OR 'adult' OR 'middle aged adults' OR 'young adult'/exp OR 'young adult' OR 'elderly'/exp OR 'elderly') AND ('random blood glucose' OR 'random blood plasma glucose' OR 'capillary blood glucose' OR 'rbg' OR 'fasting blood plasma glucose' OR 'fasting blood glucose'/exp OR 'fasting blood glucose' OR 'fbg' OR 'glycosylated hemoglobin'/exp OR 'glycosylated hemoglobin' OR 'hemoglobin a1c'/exp OR 'hemoglobin a1c' OR 'hba1c'/exp OR 'hba1c' OR 'glucose blood level'/exp OR 'glucose blood level' OR 'post prandial glucose') AND ('oral glucose tolerance test'/exp OR 'oral glucose tolerance test' OR 'ogtt'/exp OR 'ogtt') AND ('type 2 diabetes mellitus'/exp OR 'type 2 diabetes mellitus' OR 'non insulin dependent diabetes mellitus'/exp OR 'non insulin dependent diabetes mellitus' OR 'impaired glucose tolerance'/exp OR 'impaired glucose tolerance' OR 'impaired fasting glucose'/exp OR 'impaired fasting glucose' OR 'prediabetic state*' OR 'prediabet*') AND ('sensitivity'/exp OR 'sensitivity' OR 'specificity'/exp OR 'specificity' OR 'sensitivity and specificity'/exp OR 'sensitivity and specificity' OR 'predictive value'/exp OR 'predictive value' OR 'receiver operating characteristic'/exp OR 'receiver operating characteristic' OR 'diagnostic accuracy'/exp OR 'diagnostic accuracy') | 1873 |
| **SCOPUS** Inception till present  ((ALL(adults)) OR (ALL(adult*)) OR (ALL(middle-aged adult*)) OR (ALL("young adult*")) OR (ALL("frail adult*")) OR (ALL("elderly"))) AND (((ALL("Random blood glucose")) OR (ALL("Random blood plasma glucose")) OR (ALL("RBG")) OR (ALL("Random capillary blood glucose")) OR (ALL("capillary blood glucose")) OR (ALL("fasting blood glucose")) OR (ALL("fasting blood plasma glucose")) OR (ALL("FBG"))) OR (ALL("Glycated Hemoglobin A")) OR (ALL("Hb A1c")) OR (ALL("HbA1")) OR (ALL("Post Prandial Glucose")) OR (ALL("PPG")) OR (ALL("Postprandial blood glucose"))) AND ((ALL("Oral Glucose Tolerance Test")) OR (ALL("Oral Glucose Tolerance")) OR (ALL("OGTT"))) AND (((ALL("Diabetes mellitus")) OR (ALL("Type 2 Diabetes mellitus")) OR (ALL("Type II Diabetes mellitus")) OR (ALL("Non-insulin dependent diabetes mellitus")) OR (ALL("NIDDM")) OR (ALL("adult onset diabetes mellitus")) OR (ALL("prediabetic state*"))) OR (ALL("prediabetes")) OR (ALL("Impaired glucose tolerance")) OR (ALL("IGT")) OR (ALL("Impaired fasting glucose")) OR (ALL("IFG"))) AND ((ALL("Sensitivity")) OR (ALL("Specificity")) OR (ALL("Sensitivity and Specificity")) OR (ALL("likelihood ratios")) OR (ALL("predictive value of test*")) OR (ALL("ROC curve*")) OR (ALL("ROC Analys*"))) | 1843 |
| **Medline (Ovid)**  ((Aged/ or Frail Elderly/) or Adult/ or (Middle Aged/ or middle-aged adults.mp. or Aged/)) AND (diabetes mellitus.mp. or type 2 diabetes mellitus.tw. or non-insulin dependent diabetes mellitus.tw. or "prediabetic state"/ or prediabetes.tw. or "impaired glucose tolerance".tw. or "IGT".mp. or "IFG".mp. or "impaired fasting glucose".tw.) AND ("random blood glucose".tw. or "fasting blood glucose".tw. or "fasting plasma glucose".tw. or "FPG".mp. or “capillary blood glucose".tw. or "Glycated hemoglobin A"/ or “Glycated hemoglobin A".mp. or “FBG".mp. or "postprandial glucose".tw. or "HbA1c".mp. or “PPG".mp.) AND ("oral glucose tolerance test".tw. or "OGTT".mp.) AND ("diagnostic accuracy".tw. or "sensitivity".mp. or "specificity".mp. or “sensitivity and specificity"/ or "likelihood ratios".tw. or "predictive value of tests"/ or "ROC curve$".tw. or ROC curve.mp. or "ROC analys$".tw.) | 847 |
| **Cochrane**  Inception till present  (("adult"):ti,ab,kw OR MeSH descriptor: [Adult] explode all trees)) AND ("random blood glucose" OR ("random blood glucose"):ti,ab,kw OR "random plasma glucose" OR ("random plasma glucose"):ti,ab,kw OR "random capillary blood glucose" OR ("random plasma glucose"):ti,ab,kw OR "capillary blood glucose" OR ("capillary blood glucose"):ti,ab,kw OR "fasting blood glucose" OR ("fasting blood glucose"):ti,ab,kw OR "fasting blood plasma glucose" OR ("fasting blood plasma glucose"):ti,ab,kw OR "fasting capillary blood glucose" OR ("fasting capillary blood glucose"):ti,ab,kw OR "Glycated Hemoglobin A" OR ("Glycated Hemoglobin A"):ti,ab,kw OR "Hb A1c*" OR ("HbA1c*"):ti,ab,kw OR MeSH descriptor: [Glycated Hemoglobin A] explod"post prandial blood glucose" OR ("post prandial blood glucose"):ti,ab,kw) e all trees OR "post prandial glucose" OR ("post prandial glucose"):ti,ab,kw ) AND ((MeSH descriptor: [Glucose Tolerance Test] explode all trees) OR 'oral glucose tolerance test' OR ('oral glucose tolerance test'):ti,ab,kw OR "OGTT" OR ("OGTT"):ti,ab,kw) AND ('diabetes mellitus' OR ('diabetes mellitus'):ti,ab,kw OR (MeSH descriptor: [Diabetes Mellitus, Type 2] explode all trees) OR 'non-insulin dependent diabetes mellitus' OR ('non-insulin dependent diabetes mellitus'):ti,ab,kw OR "prediabet*" OR ("prediabet*"):ti,ab,kw OR MeSH descriptor: [Hyperglycemia] explode all trees AND (("Sensitivity and Specificity") OR ("Sensitivity and Specificity"):ti,ab,kw OR MeSH descriptor: [Sensitivity and Specificity] explode all trees OR "sensitivity" OR ("sensitivity"):ti,ab,kw OR "specificity" OR ("specificity"):ti,ab,kw OR "likelihood ratios" OR ("likelihood ratios"):ti,ab,kw OR MeSH descriptor: [Predictive Value of Tests] explode all trees OR "diagnostic accuracy"OR ("diagnostic accuracy"):ti,ab,kw)) | 163 |
| **CINHAL** Inception till present  (MH (adult* OR "young adult") OR (TX ("middle-aged adults OR "frail adult" OR "elderly")) AND (TX ("random blood glucose" OR "random plasma glucose" OR "capillary blood glucose" OR "RBG" OR "RCBG" OR "fasting blood glucose” OR "fasting plasma glucose" OR "FPG" OR "Glycated hemoglobin A" OR "HbA1c" OR "Post Prandial Glucose") OR MH ("Hemoglobin A, Glycosylated")) AND (MH ("Glucose Tolerance Test") OR TX("OGTT" OR "Oral Glucose Tolerance Test")) AND (MH ("Diabetes Mellitus, Type 2" OR "Prediabetic state") OR TX ("Prediabet*")) AND (MH ("Sensitivity and Specificity") OR TX ("sensitivity" OR "specificity" OR "likelihood ratio*" OR "ROC analys*")) | 75 |
| **Web of Science Core Collection** All years (1952 - 2020)  TS=("adults" OR "middle-aged adults" OR "young adults" OR "frail adults" OR "elderly") AND (TS= ("random blood glucose" OR "RBG" OR "RCBG" OR "Capillary blood glucose" OR "fasting blood glucose" OR "fasting blood plasma glucose" OR "Glycated Hemoglobin A" OR "Hb A1c" OR "HbA1 OR "Post Prandial Glucose) OR TI=("random blood glucose" OR "capillary blood glucose" OR "fasting blood glucose" OR "Glycated Hemoglobin A" OR "Hb A1c" OR "HbA1" OR "Post Prandial Glucose")) AND (TS=("Oral Glucose Tolerance Test" OR “OGTT” OR "Oral Glucose Tolerance” ) OR (TI=("Oral Glucose Tolerance Test" OR “OGTT” OR "Oral Glucose Tolerance"))) AND TS=("Diabetes mellitus" OR "Type 2 Diabetes mellitus" OR "Non-Insulin-Dependent Diabetes Mellitus" OR "NIDDM" OR "Adult-onset diabetes mellitus" OR "Prediabet*" OR "Prediabetic state" OR "Prediabetes" OR "Impaired Glucose Tolerance" OR "Impaired Fasting Glucose") OR TI=("Diabetes mellitus" OR "Type 2 Diabetes mellitus" OR "Non-Insulin-Dependent Diabetes Mellitus" OR "NIDDM" OR "Adult-onset diabetes mellitus" OR "Prediabet*" OR "Prediabetic state" OR "Prediabetes" OR "Impaired Glucose Tolerance" OR "Impaired Fasting Glucose") AND (TS=("Sensitivity" OR "Specificity" OR "Sensitivity and Specificity" OR "likelihood ratios" OR "ROC Analys*")) OR (TI=("Sensitivity" OR "Specificity" OR "Sensitivity and Specificity" OR "likelihood ratios" OR "ROC Analys*")) | 26 |

***Searches updated on March 9, 2020**

Box S2- Country (%) of Included Studies

| **Country** | **%** |
| --- | --- |
| China | 30 |
| USA | 11 |
| South Africa | 8 |
| Italy | 5 |
| Netherlands | 5 |
| Sri Lanka | 5 |
| Norway | 3 |
| India | 5 |
| Multi-country | 3 |
| Oman | 3 |
| Japan | 3 |
| Brazil | 3 |
| Singapore | 3 |
| Bangladesh | 3 |
| Thailand | 3 |
| Korea | 3 |
| Mexico | 3 |
| Australia | 3 |

**Figure S1: Year wise distribution of included studies**

**Table S2: Summary estimates for HbA1c 6.5% (WHO criteria & OGTT) for diagnosing diabetes**

|  |  | 95% Confidence Intervals | |
| --- | --- | --- | --- |
| Sensitivity | 0.520 | 0.398 | 0.639 |
| Specificity | 0.983 | 0.969 | 0.990 |
| Positive Likelihood Ratios | 29.849 | 16.568 | 53.775 |
| Negative Likelihood Ratios | 0.531 | 0.379 | 0.630 |

* Estimates are rounded off to three decimal places

**Table S3: 2 by 2 data for HBA1c studies by venous blood sample/non-capillary method of collection for diabetes**

| name | study | cutoff | TP | FP | FN | TN |
| --- | --- | --- | --- | --- | --- | --- |
| Zhou 2009 | 1 | 5.7 | 94 | 277 | 6 | 526 |
| Zhou 2009 | 1 | 6 | 80 | 81 | 20 | 722 |
| Zhou 2009 | 1 | 6.5 | 54 | 4 | 46 | 799 |
| Araneta 2010 | 2 | 6.5 | 58 | 25 | 87 | 763 |
| Kramer 2010 | 3 | 6.15 | 125 | 763 | 73 | 1146 |
| Kramer 2010 | 3 | 6.5 | 88 | 400 | 110 | 1509 |
| Riet 2010 | 4 | 5.7 | 83 | 450 | 24 | 2196 |
| Riet 2010 | 4 | 5.8 | 77 | 238 | 30 | 2408 |
| Riet 2010 | 4 | 5.9 | 72 | 159 | 35 | 2487 |
| Riet 2010 | 4 | 6 | 60 | 79 | 47 | 2567 |
| Riet 2010 | 4 | 6.1 | 45 | 53 | 62 | 2593 |
| Riet 2010 | 4 | 6.5 | 26 | 27 | 81 | 2619 |
| Riet 2010 | 4 | 7 | 13 | 0 | 94 | 2646 |
| Choi 2011 | 5 | 5 | 617 | 7735 | 18 | 1005 |
| Choi 2011 | 5 | 5.1 | 607 | 7123 | 28 | 1617 |
| Choi 2011 | 5 | 5.2 | 600 | 6301 | 35 | 2439 |
| Choi 2011 | 5 | 5.3 | 581 | 5331 | 54 | 3409 |
| Choi 2011 | 5 | 5.4 | 563 | 4318 | 72 | 4422 |
| Choi 2011 | 5 | 5.5 | 550 | 3356 | 85 | 5384 |
| Choi 2011 | 5 | 5.6 | 522 | 2473 | 113 | 6267 |
| Choi 2011 | 5 | 5.7 | 489 | 1774 | 146 | 6966 |
| Choi 2011 | 5 | 5.8 | 457 | 1206 | 178 | 7534 |
| Choi 2011 | 5 | 5.9 | 429 | 813 | 206 | 7927 |
| Choi 2011 | 5 | 6 | 393 | 568 | 242 | 8172 |
| Choi 2011 | 5 | 6.2 | 332 | 280 | 303 | 8460 |
| Choi 2011 | 5 | 6.6 | 236 | 70 | 399 | 8670 |
| Bhowmik 2013 | 6 | 5.7 | 172 | 515 | 9 | 1597 |
| Bhowmik 2013 | 6 | 6 | 156 | 141 | 25 | 1971 |
| Bhowmik 2013 | 6 | 6.5 | 138 | 40 | 43 | 2072 |
| Hui 2013 | 7 | 5.6 | 237 | 918 | 34 | 784 |
| Hui 2013 | 7 | 5.7 | 229 | 696 | 42 | 1006 |
| Hui 2013 | 7 | 5.8 | 211 | 501 | 60 | 1201 |
| Hui 2013 | 7 | 5.9 | 195 | 351 | 76 | 1351 |
| Hui 2013 | 7 | 6 | 179 | 225 | 92 | 1477 |
| Hui 2013 | 7 | 6.1 | 161 | 137 | 110 | 1565 |
| Hui 2013 | 7 | 6.2 | 149 | 82 | 122 | 1620 |
| Hui 2013 | 7 | 6.3 | 127 | 50 | 144 | 1652 |
| Hui 2013 | 7 | 6.4 | 115 | 36 | 156 | 1666 |
| Huang 2013 | 8 | 6.25 | 319 | 496 | 103 | 5622 |
| Huang 2013 | 8 | 6.4 | 302 | 300 | 120 | 5818 |
| Huang 2013 | 8 | 6.5 | 287 | 190 | 135 | 5928 |
| Vlaar 2013 | 9 | 6.3 | 22 | 36 | 13 | 873 |
| Vlaar 2013 | 9 | 6.5 | 16 | 18 | 19 | 891 |
| Liang 2014 | 10 | 5.7 | 815 | 4658 | 65 | 2701 |
| Liang 2014 | 10 | 5.8 | 794 | 3878 | 86 | 3481 |
| Liang 2014 | 10 | 5.9 | 769 | 3179 | 111 | 4180 |
| Liang 2014 | 10 | 6 | 746 | 2509 | 134 | 4850 |
| Liang 2014 | 10 | 6.1 | 712 | 1928 | 168 | 5431 |
| Liang 2014 | 10 | 6.2 | 677 | 1442 | 203 | 5917 |
| Liang 2014 | 10 | 6.3 | 635 | 1038 | 245 | 6321 |
| Liang 2014 | 10 | 6.4 | 598 | 735 | 282 | 6624 |
| Liang 2014 | 10 | 6.5 | 562 | 529 | 318 | 6830 |
| Incani 2015 | 11 | 6.5 | 20 | 30 | 31 | 381 |
| Aviles-Santa 2016 | 12 | 6.5 | 216 | 103 | 548 | 14640 |
| Hird 2016 | 13 | 5.8 | 101 | 163 | 9 | 804 |
| Hird 2016 | 13 | 5.9 | 98 | 113 | 12 | 854 |
| Hird 2016 | 13 | 6 | 98 | 77 | 12 | 890 |
| Hird 2016 | 13 | 6.1 | 92 | 41 | 18 | 926 |
| Hird 2016 | 13 | 6.3 | 83 | 30 | 27 | 937 |
| Hird 2016 | 13 | 6.5 | 77 | 12 | 33 | 955 |
| Hird 2016 | 13 | 6.7 | 74 | 6 | 36 | 961 |
| Hird 2016 | 13 | 7 | 60 | 3 | 50 | 964 |
| Karnchanasorn 2016 | 14 | 6.5 | 110 | 38 | 282 | 5334 |
| Zou 2016 | 15 | 5.8 | 146 | 240 | 23 | 1588 |
| Zou 2016 | 15 | 6 | 107 | 127 | 35 | 784 |
| Herath 2016 | 16 | 6.5 | 32 | 38 | 9 | 175 |
| Zhou 2018 | 17 | 5.8 | 484 | 1596 | 211 | 5618 |
| Zhou 2018 | 17 | 5.9 | 461 | 1300 | 234 | 5914 |
| Zhou 2018 | 17 | 6 | 442 | 1092 | 253 | 6122 |
| Zhou 2018 | 17 | 6.1 | 412 | 190 | 283 | 7024 |
| Zhou 2018 | 17 | 6.5 | 294 | 398 | 401 | 6816 |
| Lim 2018 | 18 | 5.7 | 306 | 1598 | 26 | 1610 |
| Lim 2018 | 18 | 5.8 | 299 | 1188 | 33 | 2031 |
| Lim 2018 | 18 | 5.9 | 291 | 773 | 41 | 2435 |
| Lim 2018 | 18 | 6 | 283 | 472 | 49 | 2736 |
| Lim 2018 | 18 | 6.1 | 274 | 311 | 58 | 2897 |
| Lim 2018 | 18 | 6.2 | 248 | 176 | 84 | 3032 |
| Lim 2018 | 18 | 6.3 | 225 | 99 | 107 | 3109 |
| Lim 2018 | 18 | 6.4 | 193 | 67 | 139 | 3141 |
| Lim 2018 | 18 | 6.5 | 169 | 38 | 163 | 3170 |
| Lim 2018 | 18 | 7 | 111 | 6 | 221 | 3202 |
| Prakashchandra 2018 | 19 | 6.15 | 120 | 318 | 34 | 906 |
| Prakashchandra 2018 | 19 | 6.25 | 116 | 245 | 38 | 979 |
| Prakashchandra 2018 | 19 | 6.3 | 109 | 184 | 45 | 1040 |
| Prakashchandra 2018 | 19 | 6.4 | 106 | 159 | 48 | 1065 |
| Prakashchandra 2018 | 19 | 6.5 | 104 | 122 | 50 | 1102 |
| Wu 2017 | 20 | 6.5 | 212 | 38 | 385 | 3690 |
| Mohan 2010 | 21 | 6.1 | 195 | 240 | 25 | 1728 |

**Table S4: 2 by 2 data for FPG studies for diabetes**

| Name of study & year | Cut-off | True Positive | False Positive | False Negative | True Negative |
| --- | --- | --- | --- | --- | --- |
| Moran 2001 | 110 | 64 | 48 | 1 | 599 |
| Moran 2001 | 126 | 39 | 37 | 26 | 610 |
| Daniel 2002 | 126 | 273 | 46 | 104 | 2826 |
| Daniel 2002 | 140 | 232 | 18 | 145 | 2854 |
| Mannucci 2003 | 126 | 75 | 193 | 5 | 942 |
| Nakagami 2003 | 104 | 826 | 2469 | 225 | 13992 |
| Nakagami 2003 | 126 | 478 | 230 | 573 | 16231 |
| Nakagami 2003 | 140 | 326 | 66 | 725 | 16395 |
| Al-Lawati 2006 | 106 | 428 | 407 | 61 | 4021 |
| Al-Lawati 2006 | 126 | 334 | 61 | 155 | 4367 |
| Huang 2015 | 126 | 96 | 85 | 135 | 5466 |
| Aekplahorn 2015 | 126 | 313 | 0 | 446 | 6125 |
| Herath 2017 | 126 | 27 | 4 | 14 | 209 |
| Prakashchandra 2018 | 99 | 126 | 233 | 28 | 991 |
| Prakashchandra 2018 | 100 | 122 | 184 | 32 | 1040 |
| Prakashchandra 2018 | 102.6 | 117 | 135 | 37 | 1089 |
| Prakashchandra 2018 | 104.5 | 112 | 122 | 42 | 1102 |
| Prakashchandra 2018 | 106.2 | 108 | 86 | 46 | 1138 |
| Prakashchandra 2018 | 126 | 62 | 12 | 92 | 212 |
| Katulanda 2019 | 79.2 | 184 | 2695 | 7 | 1128 |
| Katulanda 2019 | 90 | 174 | 1120 | 17 | 2703 |
| Katulanda 2019 | 95 | 168 | 597 | 23 | 3226 |
| Katulanda 2019 | 100 | 154 | 302 | 37 | 3521 |
| Katulanda 2019 | 110 | 118 | 77 | 73 | 3746 |
| Katulanda 2019 | 126 | 90 | 16 | 101 | 3807 |

Figure S2: Quality Assessment of each study with HBA1c as index test for diabetes and prediabetes


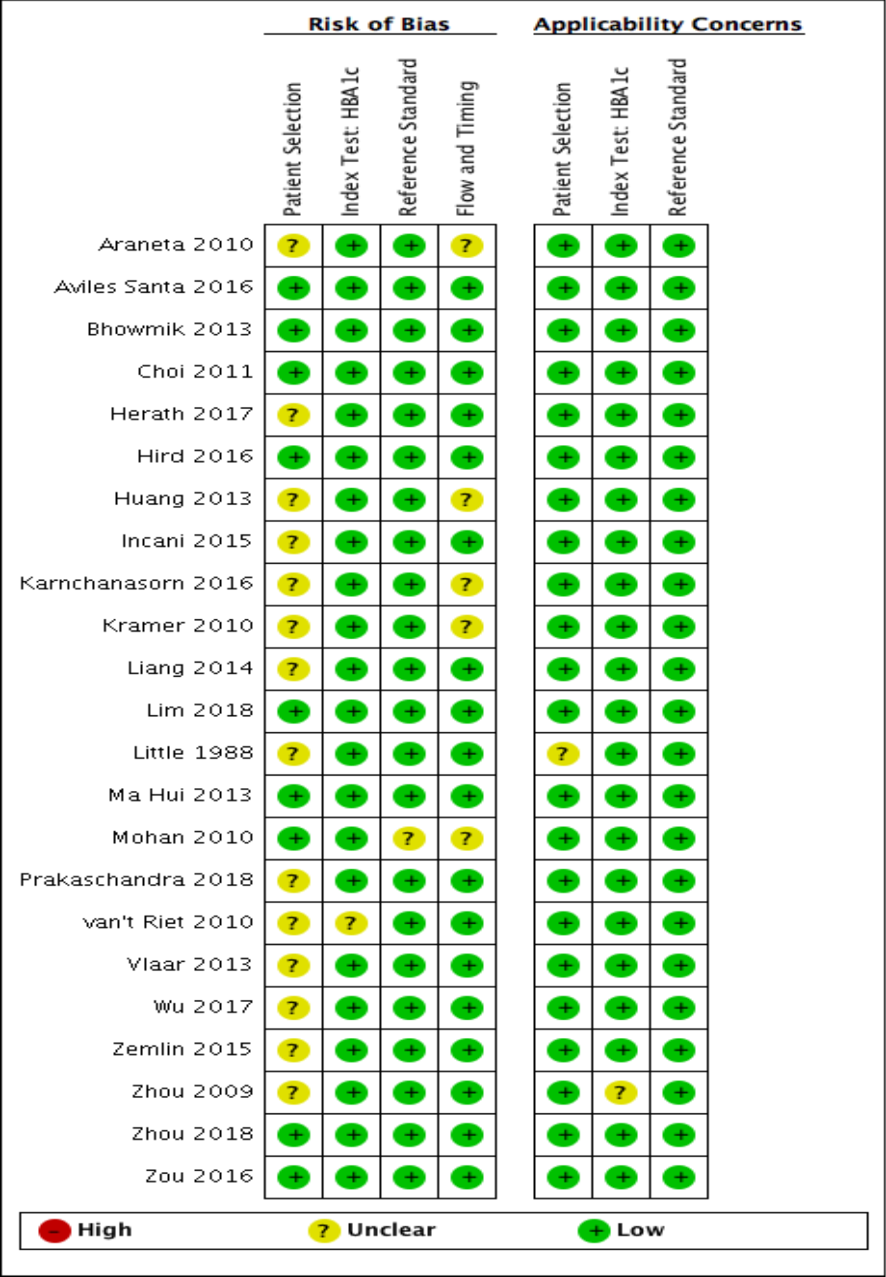


Figure S3: Risk of bias graph for HbA1c (n=23) for diabetes and prediabetes


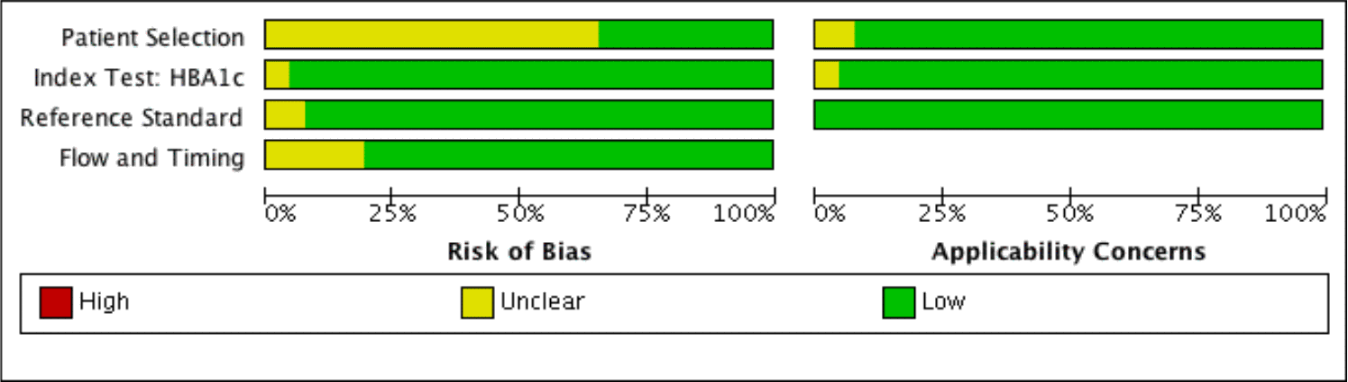


Figure S4: Quality Assessment of each study with HBA1c (capillary) as index test


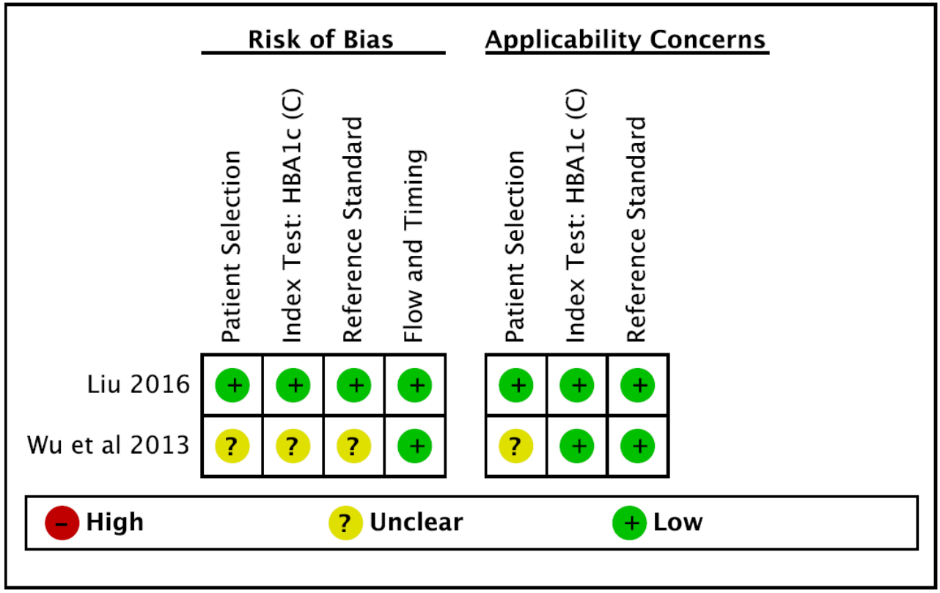


S5 Fig5: Quality Assessment of each study with FPG as index test (n=13)


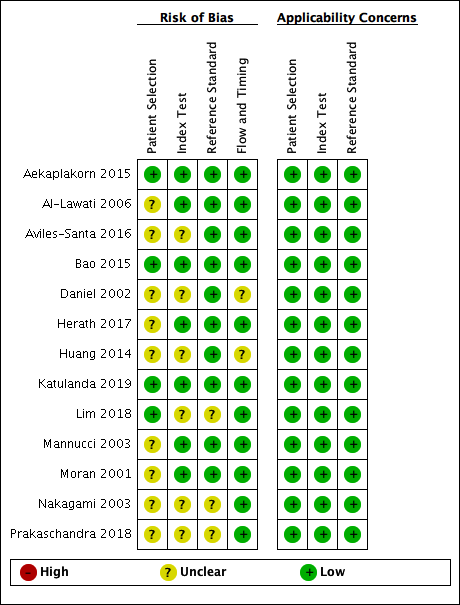


Figure S6: Risk of bias graph for FPG (n=13)

**
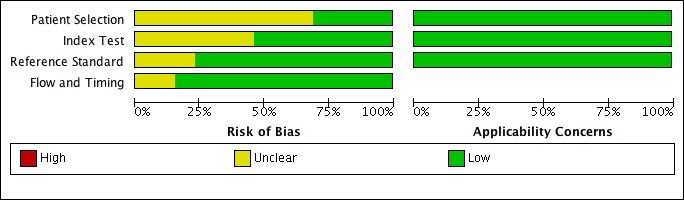
**

**GRADE Evidence Profile Tables for Index HbA1c Optimal cut-off 6.03% for diabetes (2)**

**Table S5:** Should HbA1c 6.03% be used to diagnose type 2 diabetes in previously undiagnosed adults?

| \| Sensitivity \| 0.74 (95% CI: 0.68 to 0.79) \| \| --- \| --- \| \| Specificity \| 0.87 (95% CI: 0.82 to 0.91) \| |  | \| Prevalences* \| 6.77% \| 9.38% \| 11.07% \| \| --- \| --- \| --- \| --- \| |
| --- | --- | --- | --- | --- | --- | --- | --- | --- | --- | --- |

| Outcome | № of studies (№ of patients) | Study design | Factors that may decrease certainty of evidence | | | | | Effect per 1,000 patients tested | | | Test accuracy CoE |
| --- | --- | --- | --- | --- | --- | --- | --- | --- | --- | --- | --- |
|  |  |  | Risk of bias | Indirectness | Inconsistency | Imprecision | Publication bias | pre-test probability of 6.77% | pre-test probability of 9.38% | pre-test probability of 11.07% |  |
| **True positives** (patients with [target condition]) | 21 studies 6388 patients | cross-sectional (cohort type accuracy study) | serious ^a^ | not serious | not serious | not serious | none | 50 (46 to 54) | 69 (64 to 74) | 82 (75 to 88) | ⨁⨁⨁◯ MODERATE |
| **False negatives** (patients incorrectly classified as not having [target condition]) |  |  |  |  |  |  |  | 18 (14 to 22) | 25 (20 to 30) | 29 (23 to 36) |  |
| **True negatives** (patients without [target condition]) | 21 studies 73841 patients | cross-sectional (cohort type accuracy study) | serious ^a^ | not serious | not serious | not serious | none | 813 (764 to 848) | 790 (743 to 825) | 775 (729 to 809) | ⨁⨁⨁◯ MODERATE |
| **False positives** (patients incorrectly classified as having [target condition]) |  |  |  |  |  |  |  | 119 (84 to 168) | 116 (81 to 163) | 114 (80 to 160) |  |

* The number of true and false positives and negatives are calculated for a hypothetical cohort of 1,000 at 25^th^,50^th^ (median) and 75^th^ percentile prevalence calculated from 21 studies included for HbA1c.

Explanations

1. Lack on details on sampling (random/consecutive) of participants in many included studies leading to unclear risk in patient selection in QUADAS-2

Judgement of the certainty of evidence was done based on the following (2-4):

**Risk of bias (3):**

If all risk of bias items were at low risk according to the QUADAS 2 tool then no downgrading of evidence was done. The domain was assessed as not serious. However, there was any unclear or high-risk sub-domain in the QUADAS-2 then accordingly serious or very serious rating of certainty of evidence was considered.

**Indirectness:** Where the execution/methods of collection of the index test or reference test were not clearly mentioned in the study, indirectness was rated as serious.

**Inconsistency:** All available information on thresholds was used instead of selective pair of sensitivity and specificity then not serious

**Imprecision:** If confidence intervals around summary estimates of sensitivity and specificity are wide or small number of participants then serious limitation was considered.

**Table S6: Details on 2 ×2 table for various cut-offs for prediabetes in included studies**

| Author | Year | DIAGNOSTIC CRITERIA | Cut off for Blood Glucose Test | True Positive | False Positive | False negative | True Negative |
| --- | --- | --- | --- | --- | --- | --- | --- |
| Zhou | 2018 | WHO | 5.5 | 860 | 2155 | 654 | 4240 |
|  |  |  |  |  |  |  |  |
| LIANG | 2014 | WHO | 5.6 | 1326 | 4652 | 239 | 2022 |
| Zhou | 2018 |  | 5.6 | 784 | 1750 | 730 | 4645 |
|  |  |  |  |  |  |  |  |
| LIANG | 2014 | WHO | 5.7 | 1230 | 3944 | 335 | 2730 |
| ZHOU ET AL | 2009 | WHO | 5.7 | 120 | 183 | 82 | 518 |
| Zhou | 2018 | WHO | 5.7 | 679 | 1346 | 835 | 5049 |
|  |  |  |  |  |  |  |  |
| ZEMLIN | 2015 | ADA | 5.75 | 113 | 173 | 72 | 309 |
|  |  |  |  |  |  |  |  |
| LIANG | 2014 | WHO | 5.8 | 1114 | 3177 | 451 | 349 |
| Hui | 2013 | WHO | 5.8 | 213 | 499 | 261 | 1000 |
|  |  |  |  |  |  |  |  |
| LIANG | 2014 | WHO | 5.9 | 1009 | 2436 | 556 | 4238 |
|  |  |  |  |  |  |  |  |
| LIANG | 2014 | WHO | 6 | 876 | 1883 | 689 | 4791 |
| ZHOU ET AL | 2009 | WHO | 6 | 51 | 36 | 151 | 665 |
|  |  |  |  |  |  |  |  |
| LIANG | 2014 | WHO | 6.1 | 753 | 1369 | 812 | 5305 |
|  |  |  |  |  |  |  |  |
| LIANG | 2014 | WHO | 6.2 | 626 | 941 | 939 | 5733 |
|  |  |  |  |  |  |  |  |
| LIANG | 2014 | WHO | 6.3 | 494 | 628 | 1071 | 6046 |
|  |  |  |  |  |  |  |  |
| LIANG | 2014 | WHO | 6.4 | 382 | 407 | 1183 | 6267 |
|  |  |  |  |  |  |  |  |
| LIANG | 2014 | WHO | 6.5 | 293 | 274 | 1272 | 6400 |
| ZHOU ET AL | 2009 | WHO | 6.5 | 4 | 0 | 198 | 701 |
| VIAAR | 2013 | ADA | 6.5 | 126 | 241 | 65 | 512 |
|  |  |  |  |  |  |  |  |
| INCANI | 2015 | ADA | 5.7-6.4 | 160 | 49 | 140 | 113 |
|  |  |  |  |  |  |  |  |
| VIAAR | 2013 | ADA | 5.8-6.3 | 111 | 166 | 80 | 587 |
|  |  |  |  |  |  |  |  |
| LIU | 2016 | WHO | 5.1 | 464 | 6740 | 9 | 398 |
|  |  |  | 5.2 | 458 | 6478 | 15 | 660 |
|  |  |  | 5.3 | 444 | 6037 | 29 | 1101 |
|  |  |  | 5.4 | 428 | 5450 | 45 | 1688 |
|  |  |  | 5.5 | 407 | 4819 | 66 | 2319 |
|  |  |  | 5.6 | 379 | 4113 | 94 | 3025 |
|  |  |  | 5.7 | 346 | 3348 | 127 | 3790 |
|  |  |  | 5.8 | 306 | 2550 | 167 | 4588 |
|  |  |  | 5.9 | 256 | 1834 | 217 | 5304 |
|  |  |  | 6.0 | 206 | 1278 | 267 | 5860 |
|  |  |  | 6.1 | 164 | 901 | 309 | 6237 |
|  |  |  | 6.2 | 124 | 579 | 349 | 6559 |
|  |  |  | 6.3 | 90 | 361 | 383 | 6777 |
|  |  |  | 6.4 | 61 | 213 | 412 | 6925 |
|  |  |  | 6.5 | 43 | 128 | 430 | 7010 |
|  |  |  | 6.6 | 27 | 78 | 446 | 7060 |
|  |  |  | 6.7 | 17 | 42 | 456 | 7096 |
|  |  |  | 6.8 | 12 | 19 | 461 | 7119 |
|  |  |  | 6.9 | 8 | 15 | 465 | 7123 |
|  |  |  | 7 | 5 | 11 | 468 | 7127 |
|  |  |  | 7.1 | 3 | 10 | 470 | 7128 |
|  |  |  | 7.2 | 2 | 8 | 471 | 7130 |

**References**

1. Adamska E, Waszczeniuk M, Goscik J, Golonko A, Wilk J, Pliszka J, et al. The usefulness of glycated hemoglobin A1c (HbA1c) for identifying dysglycemic states in individuals without previously diagnosed diabetes. Advances in medical sciences. 2012;57(2):296-301.

2. University M. GRADEpro GDT: GRADEpro Guideline Development Tool [Software]., 2015 . : (developed by Evidence Prime, Inc.); 2015 [Available from: gradepro.org.

3. Schünemann HJ, Mustafa R, Brozek J, Santesso N, Alonso-Coello P, Guyatt G, et al. GRADE Guidelines: 16. GRADE evidence to decision frameworks for tests in clinical practice and public health. Journal of clinical epidemiology. 2016;76:89-98.

4. Caliesch R, Sattelmayer M, Reichenbach S, Zwahlen M, Hilfiker R. Diagnostic accuracy of clinical tests for cam or pincer morphology in individuals with suspected FAI syndrome: a systematic review. BMJ open sport & exercise medicine. 2020;6(1):e000772.
